# Protective Effects of Cilastatin against Vancomycin-Induced Nephrotoxicity

**DOI:** 10.1155/2015/704382

**Published:** 2015-10-04

**Authors:** Blanca Humanes, Juan Carlos Jado, Sonia Camaño, Virginia López-Parra, Ana María Torres, Luís Antonio Álvarez-Sala, Emilia Cercenado, Alberto Tejedor, Alberto Lázaro

**Affiliations:** ^1^Renal Physiopathology Laboratory, Department of Nephrology, Gregorio Marañón University Hospital, IiSGM, 28007 Madrid, Spain; ^2^Department of Medicine, School of Medicine, Complutense University of Madrid, 28040 Madrid, Spain; ^3^Department of Internal Medicine, Gregorio Marañón University Hospital, IiSGM, 28007 Madrid, Spain; ^4^Clinical Microbiology and Infectious Diseases Department, Gregorio Marañón University Hospital, IiSGM, 28007 Madrid, Spain

## Abstract

Vancomycin is a very effective antibiotic for treatment of severe infections. However, its use in clinical practice is limited by nephrotoxicity. Cilastatin is a dehydropeptidase I inhibitor that acts on the brush border membrane of the proximal tubule to prevent accumulation of imipenem and toxicity. The aim of this study was to investigate the potential protective effect of cilastatin on vancomycin-induced apoptosis and toxicity in cultured renal proximal tubular epithelial cells (RPTECs). Porcine RPTECs were cultured in the presence of vancomycin with and without cilastatin. Vancomycin induced dose-dependent apoptosis in cultured RPTECs, with DNA fragmentation, cell detachment, and a significant decrease in mitochondrial activity. Cilastatin prevented apoptotic events and diminished the antiproliferative effect and severe morphological changes induced by vancomycin. Cilastatin also improved the long-term recovery and survival of RPTECs exposed to vancomycin and partially attenuated vancomycin uptake by RPTECs. On the other hand, cilastatin had no effects on vancomycin-induced necrosis or the bactericidal effect of the antibiotic. This study indicates that cilastatin protects against vancomycin-induced proximal tubule apoptosis and increases cell viability, without compromising the antimicrobial effect of vancomycin. The beneficial effect could be attributed, at least in part, to decreased accumulation of vancomycin in RPTECs.

## 1. Introduction

Vancomycin (VAN) is a glycopeptide antibiotic that is widely used for the treatment of severe Gram-positive infections such as those caused by methicillin-resistant* Staphylococcus aureus* (MRSA) and* Staphylococcus epidermidis* [1, 2].

Patients hospitalized in the cardiac care or cardiovascular surgery units frequently require an intravenous or intra-arterial catheter. Approximately 3% of these patients develop catheter-related bloodstream infection (CRBSI), although the incidence may be as high as 16% [3]. In clinical cases of prolonged* S. aureus* CRBSI, VAN is the most commonly used antimicrobial treatment [4]. Nevertheless, VAN has potentially fatal side effects [1, 2, 5, 6]. Nephrotoxicity is the side effect that most limits the dose of VAN, particularly in patients receiving high doses or combinations with other antibiotics, such as aminoglycosides [7]. VAN-induced nephrotoxicity has been reported to occur in 5–25% of patients [2, 8], although this incidence can rise to 20–35%, with a consequent increase in the severity of renal failure when VAN is administered concomitantly with aminoglycosides [9].

The mechanism underlying VAN-induced nephrotoxicity remains unclear despite numerous studies performed over several decades, although some authors have suggested that it is similar to that of gentamicin [10]. Recent animal and cellular studies have shown that oxidative stress, inflammatory events, and apoptotic cell death might play a role in the pathogenesis of VAN-induced nephrotoxicity [1, 2, 7], which directly affects renal proximal tubular epithelial cells (RPTECs) and leads to renal tubular ischemia and acute tubulointerstitial damage [2, 8, 11]. In fact, increased urinary excretion of proximal tubule cells after administration of VAN has been demonstrated in animal studies [12]. VAN directly triggers depolarization of mitochondrial membrane potential, release of cytochrome c, and activation of caspase 9, which in turn activates caspase 3, a key component in the execution stage of apoptosis [7].

Prevention of VAN-induced nephrotoxicity without decreasing efficacy is a highly desirable objective in treatment of MRSA-induced CRBSI. Although several* in vitro* and* in vivo* approaches have been proposed to reduce VAN-induced renal toxicity, such as antioxidants or erythropoietin [1, 7, 11, 13, 14], it is unclear whether such approaches would limit the bactericidal capacity of VAN. Therefore applicability in humans is questionable and has yet to be established [15]. Therapeutic drug monitoring is one of the few effective options for prevention of VAN-induced nephrotoxicity, although it is clearly insufficient [2, 7], and the search for alternative protective strategies against toxic damage to the proximal tubule is a key research area today.

We previously reported the usefulness of cilastatin in the prevention of acute kidney injury (AKI) induced by common nephrotoxic agents (e.g., cisplatin) without reducing therapeutic activity [16–20]. Cilastatin is an inhibitor of dehydropeptidase I (DHP-I), which is found in the cholesterol rafts of the brush border of RPTECs [18]. Our experimental evidence suggests that binding of cilastatin to DHP-I interacts with apical cholesterol lipid rafts [16, 18, 19] to protect (*in vivo* and* in vitro)* against the apoptosis and oxidative stress induced by nephrotoxic agents. Clinical studies also support this protective role of cilastatin (imipenem-cilastatin) against cyclosporine A- (CsA-) induced nephrotoxicity [21–24].

Studies have shown that cilastatin (or imipenem-cilastatin) has the potential to protect against VAN-induced nephrotoxicity [25–27]; however, evidence for the antiapoptotic effects of cilastatin on VAN-induced AKI is insufficient. Thus, the aims of the present study were to evaluate the role of cell death as the main pathogenic mechanism in VAN-mediated renal cell injury and to evaluate whether cilastatin can reduce or prevent VAN-induced proximal tubule cell death without compromising bactericidal power.

## 2. Material and Methods

### 2.1. Chemicals

VAN was obtained from Normon (Madrid, Spain) and dissolved in cell culture medium at the specified concentrations.

Crystalline cilastatin was kindly provided by Merck Sharp & Dohme S.A. (Madrid, Spain). A dose of 200 *μ*g/mL was chosen because it is cytoprotective and falls within the reference range for clinical use [18, 19].

### 2.2. Proximal Tubular Primary Cell Culture

Porcine RPTECs were obtained as previously described [18]. Briefly, cortex was obtained by slicing a kidney and disaggregated by incubation in Ham's F-12 medium containing collagenase A (Boehringer Mannheim, Germany) at a final concentration of 0.6 mg/mL. Digested tissue was then filtered, washed, and centrifuged by resuspension in isotonic, sterile Percoll gradient (45% [v/v]) at 20,000 g for 30 minutes. Proximal tubules were collected from the deepest fraction, washed, and resuspended in supplemented DMEM/Ham's F-12 in a 1 : 1 ratio (with 25 mM HEPES, 3.7 mg/mL sodium bicarbonate, 2.5 mM glutamine, 1% nonessential amino acids, 100 U/mL penicillin, 100 mg/mL streptomycin, 5 × 10^−8^ M hydrocortisone, 5 mg/mL ITS, and 2% fetal bovine serum). Proximal tubules were seeded at a density of 0.66 mg/mL and incubated at 37°C in a 95% air/5% CO_2_ atmosphere. RPTECs were used when they reached confluence (~80%).

### 2.3. Cell Morphology Analysis

Pictures of cell morphology were obtained using 4x objective of Olympus IX70 microscope (Olympus, Hamburg, Germany) in phase-contrast imaging 24 hours after treatment with VAN (0.6, 3, and 6 mg/mL) or VAN plus cilastatin (200 *μ*g/mL).

### 2.4. Quantification of Cell Detachment

RPTECs were cultured and treated with VAN (0.6, 3, and 6 mg/mL) in the presence or absence of cilastatin (200 *μ*g/mL) for 24 h. Detached cells were collected, resuspended in 300 *μ*L of phosphate-buffered saline (PBS), and quantified by flow cytometry (Gallios Beckman Coulter, Barcelona, Spain). Results were obtained as cell counting for 60 s and we selected the gate according to FS (forward scatter) and SS (side scatter). These data were analyzed using Kaluza for Gallios Software (Beckman Coulter).

### 2.5. Measurement of Apoptosis and Necrosis

Cell nuclei were visualized after DNA staining with the fluorescent dye 4,6-diamidino-2-phenylindole (DAPI, Sigma-Aldrich, Missouri, USA) in order to detect evidence of apoptosis. In brief, cells on coverslips were treated with VAN (0.6, 3, or 6 mg/mL) with or without cilastatin 200 *μ*g/mL for 24 h. Thereafter, cells were fixed in 4% formaldehyde for 10 min, rinsed with PBS, and permeabilized with 0.5% Triton X-100 for 5 min. Cells were then rinsed with PBS and incubated with DAPI (12.5 *μ*g/mL) at room temperature for 15 min. Finally after removing excess dye, coverslips were mounted in microscope slides and imaging was performed as previously described [18, 20].

DNA fragmentation was measured in RPTECs treated with VAN (0.6, 3, or 6 mg/mL) in the presence or not of cilastatin 200 *μ*g/mL using Cell Death Detection ELISA^PLUS^ Kit (Boehringer Mannheim, Roche, Mannheim, Germany) according to the manufacturer's protocol.

To detect any evidence of necrosis, release of lactate dehydrogenase (LDH) from RPTECs was measured in the culture medium 24 and 48 h after exposure to VAN (3 and 6 mg/mL) in the presence or not of cilastatin (200 *μ*g/mL), as previously described [18]. Release of LDH was expressed relative to total LDH released by treatment with 0.1% Triton X-100 (100% release).

### 2.6. Measurement of Early/Late Apoptosis Using Flow Cytometry

Early and late VAN-induced apoptosis were measured using annexin V (BD Pharmingen, Madrid, Spain) and propidium iodide (PI, Sigma-Aldrich). Cells were pretreated with VAN (0.6, 3, and 6 mg/mL) alone or in combination with cilastatin (200 *μ*g/mL) before being trypsinized, washed twice with PBS, and incubated for 30 min in the dark in 100 *μ*L buffer containing 5 *μ*L fluorescein isothiocyanate- (FITC-) labeled annexin V and 5 *μ*L PI for flow cytometry (Gallios, Beckman Coulter). At least 10 000 cells were analyzed in each case. Data were analyzed using Kaluza for Gallios Software (Beckman Coulter).

### 2.7. Cell Viability Assay

Cell survival was measured by MTT assay as described previously [18, 20]. In brief, after 24 h treatment with VAN 0.6, 3, or 6 mg/mL alone or in combination with cilastatin (200 *μ*g/mL), RPTECs were incubated with 0.5 mg/mL of MTT for 3 h in darkness at 37°C. Thereafter, the volume was removed and 100 *μ*L of 50% dimethylformamide in 20% SDS (pH 4.7) was added, incubating plates at 37°C overnight. The amount of colored formazan formed was measured at 595 nm.

Alternatively, an Olympus IX70 inverted microscope fitted to a spectrofluorometer SLM AMINCO 2000 was used to measure MTT reduction in real time on single cells at 570 nm, as previously described [20]. Recordings of the first seconds after addition of MTT show the initial kinetics of MTT reduction and formazan production, thus offering a first approach to the activity and function of the mitochondrial chain in intact cells.

### 2.8. Quantification of Colony-Forming Units

RPTECs were plated on six-well plates and treated for 24 h with VAN 3 or 6 mg/mL alone or in combination with cilastatin (200 *μ*g/mL), to measure the long-term protective effects of cilastatin as described previously [18, 20]. Briefly, supernatants were discarded and adherent cells were washed in saline serum, trypsinized, seeded in Petri dishes (100 mm), and allowed to grow for 7 days in drug-free complete medium. Adherent cells colonies were fixed for 5 minutes with 5% paraformaldehyde/PBS and stained with 0.5% crystal violet/20% methanol for 2 minutes. Excess dye was removed by washing with PBS. Finally, crystal violet was eluted with 50% ethanol/50% sodium citrate 0.1 M (pH 4.2) and quantified at 595 nm.

### 2.9. Cellular VAN Transport and Accumulation

Accumulation of VAN in RPTECs was measured using a Fluorescence Polarization Immunoassay technology on a TDX Chemistry Analyzer (Abbot Laboratories, USA) in accordance with the manufacturer's instructions, in the same way that it was described previously [20]. The results were expressed as follows: [*μ*g VAN/*μ*g protein].

### 2.10. Microorganism Susceptibility Assays

We tested 8 unique clinical isolates collected from blood, abscesses, and urine from patients in our hospital in 2012. The isolates corresponded to 4* Staphylococcus aureus* strains (2 methicillin-susceptible and 2 methicillin-resistant), 3* Enterococcus faecalis* strains, and 1* Enterococcus faecium* strain. Previous minimum inhibitory concentration (MIC) based on microdilution testing (MicroScan panels, Siemens, Sacramento, USA) revealed that all Gram-positive isolates were susceptible to VAN.

*Susceptibility Testing*. To determine MICs broth microdilution method was performed with standard cation-adjusted Mueller-Hinton broth (CAMHB) as previously described in the guidelines of the Clinical and Laboratory Standards Institute [28]. VAN was tested at dilutions ranging from 0.06 to 64 *μ*g/mL with or without cilastatin (200 *μ*g/mL).

Minimum bactericidal concentrations (MBCs) were determined as previously described [29, 30]. Briefly, 0.1 mL from the MIC well and 4 further dilutions were cultured in blood agar plates and incubated at 37°C for 24 to 48 h. The values of MBCs were recorded as the lowest dilution decreasing ≥99.9 in growth (≥3-log⁡10 reduction in colony-forming units (CFU)/mL) in comparison with control.

We compared the results obtained with VAN alone or in combination with cilastatin.

### 2.11. Statistical Methods

Quantitative variables were summarized as the mean ± standard error of the mean (SEM). Differences were considered statistically significant for bilateral alpha values under 0.05. Factorial ANOVA was used when more than 1 factor was considered. When a single factor presented more than 2 levels, a post hoc analysis (least significant difference) was performed, if the model showed significant differences between factors. When demonstrative results are shown, they represent a minimum of at least 3 repeats. When possible, a quantification technique was used to illustrate reproducibility.

## 3. Results

### 3.1. Cilastatin Reduces VAN-Induced Proximal Tubular Cell Damage

**Figure 1:**
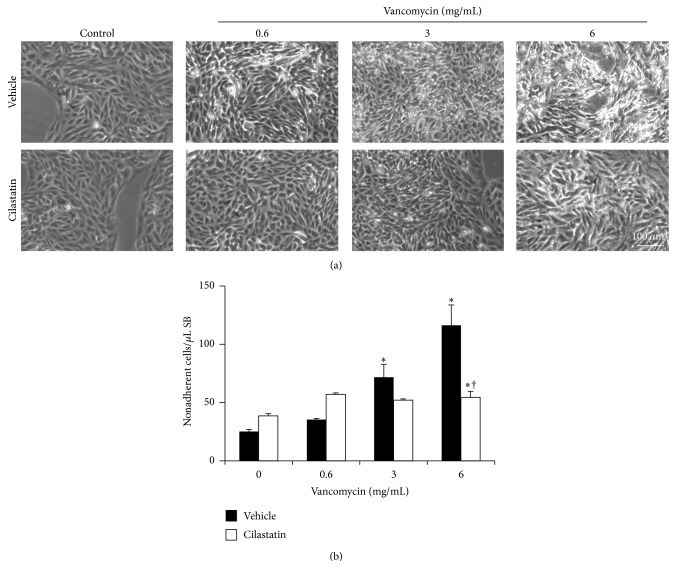
Effects of cilastatin on vancomycin-treated renal proximal tubular epithelial cells (RPTECs) morphology. RPTECs were cultured in the presence of vancomycin (0.6, 3, and 6 mg/mL) and vancomycin plus cilastatin (200 *μ*g/mL) for 24 hours. (a) Phase-contrast photomicrographs are shown (representative example of at least three independent experiments; original magnification 40x). (b) Effect of cilastatin on vancomycin-induced detachment of RPTECs, measured by flow cytometry and determined by counting the number of cells in an equal volume of buffer. Data are represented as the mean ± SEM of at least three separate experiments. ^*∗*^ *p* ≤ 0.05 versus control and control plus cilastatin, ^†^ *p* ≤ 0.0001 versus the same data without cilastatin.

VAN induces dose-dependent cell death in primary culture of RPTECs. When RPTECs are exposed to increasing concentrations of VAN for 24 hours, direct observation by phase microscopy shows cell rounding and detachment from the plate. Cilastatin significantly reduced the impact observed at every VAN concentration (Figure 1(a)).

However, VAN-induced cell death causes early detachment of damaged cells from the plate.  Figure 1(b) shows the quantification of nonadherent cells from control plates and VAN-treated plates (0.6, 3, and 6 mg/mL) in combination or not with cilastatin. Cilastatin significantly reduced cell detachment in cells treated with 3 and 6 mg/mL.

### 3.2. Cilastatin Protects against VAN-Induced Apoptosis but Not Necrosis

**Figure 2:**
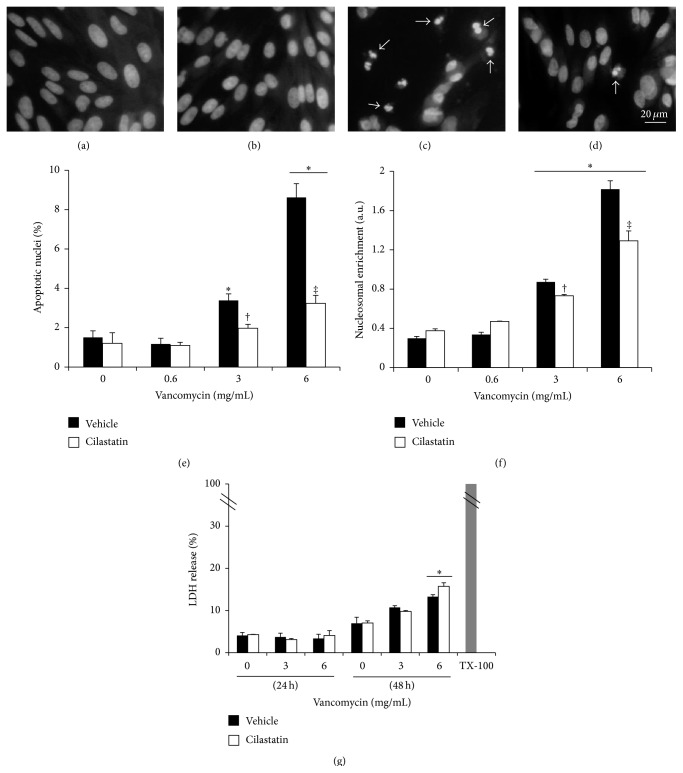
Cilastatin protects against vancomycin-induced apoptosis but not necrosis. Proximal tubular epithelial cells (RPTECs) were cultured in the presence of vancomycin (0.6 and/or 3 and 6 mg/mL) and vancomycin plus cilastatin (200 *μ*g/mL) for 24 and/or 48 hours. (a–d) Nuclear staining with DAPI. Adherent RPTECs were stained with DAPI to study if apoptotic-like nuclear morphology was present. Arrows point to fragmented apoptotic nuclei. (e) Quantitative approach to the images presented in (a–d). (f) Oligonucleosomes at 24 hours were quantified in the cell soluble fraction and detected with an ELISA kit. (g) Effect of cilastatin in vancomycin-induced release of LDH. Data are presented as % of total release of LDH obtained by Triton X 100 (TX-100) cell treatment. Data are represented as the mean ± SEM of at least three separate experiments. ^*∗*^ *p* < 0.007 versus control and control plus cilastatin, ^†^ *p* ≤ 0.05 versus the same data without cilastatin, ^‡^ *p* < 0.0001 versus the same data without cilastatin.

Estimation of apoptotic cell death was obtained in adherent cells stained with DAPI (Figures 2(a)–2(d)). Incubation with 0.6, 3, and 6 mg/mL led to cell shrinkage with significant nuclear condensation, fragmentation, and formation of apoptotic-like bodies (arrows).  Figure 2(e) shows quantification of apoptotic nuclei in adherent cells. Treatment with cilastatin significantly ameliorates VAN-induced nuclear apoptosis.

After 24 hours of exposure to VAN 0.6, 3, and 6 mg/mL, apoptosis of RPTECs measured as nucleosomal DNA fragmentation and migration from nuclei to cytosol was quantified and compared with apoptosis under the same conditions but in the presence of cilastatin (Figure 2(f)). RPTECs exposed to 3 and 6 mg/mL VAN present an increase in nucleosomes recovered from cytosol. Cilastatin significantly prevented these changes in nucleosomal enrichment.

To evaluate the effect of cilastatin on VAN-induced necrosis, release of LDH from RPTECs to the culture medium was measured after treatment with VAN 3 and 6 mg/mL in combination or not with cilastatin at different time periods. After 24 hours no changes were found in LDH values at any concentration of VAN, and slight changes were found after 48 h only with VAN 6 mg/mL (≤5% of maximal release of LDH). Interestingly, coincubation with cilastatin did not modify this small increase in necrotic cell death. Thus, reduction of VAN-induced cell death with cilastatin seems to be specific for apoptosis (Figure 2(g)).

### 3.3. Cilastatin Protects against VAN-Induced Early and Late Apoptosis

To evaluate the effect of cilastatin on VAN-induced early and late apoptosis, RPTECs stained with annexin V and PI were analyzed after treatment with VAN (0.6, 3, and 6 mg/mL) with or without cilastatin (200 *μ*g/mL) for 24 h.

**Figure 3:**
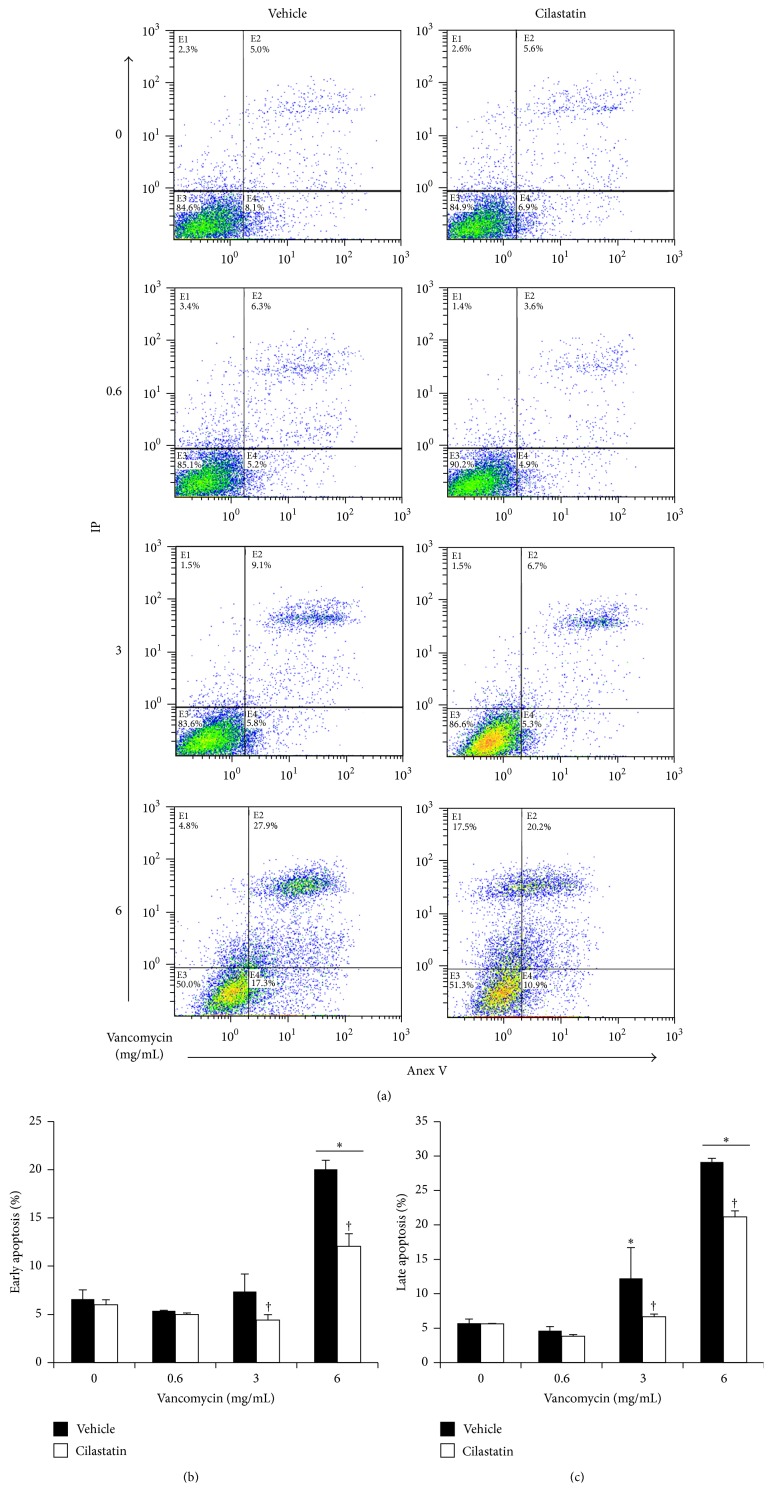
Effect of cilastatin on vancomycin-induced early and late apoptosis. Vancomycin-induced early and late-apoptotic cell death in proximal tubular epithelial cells and the effect of cilastatin were determined by flow cytometry with annexin V/propidium iodide assay after 24 hours of treatments. (a) Representative scatter plots of propidium iodide (*y* axis) versus annexin V (*x* axis). The lower right quadrants represent the early-apoptotic cells (annexin V-positive/propidium iodide-negative) and the upper right quadrants represent the late-apoptotic cells (annexin V-positive/propidium iodide-positive). (b) Quantification of early-apoptotic cells in all conditions (lower right quadrants). (c) Quantification of late-apoptotic cells in all conditions (upper right quadrants). Results are expressed as % of total cells quantified. Data are represented as the mean ± SEM of at least three separate experiments. ^*∗*^ *p* < 0.05 versus control and control plus cilastatin, ^†^ *p* < 0.05 versus the same data without cilastatin. IP, propidium iodide. Anex V, annexin V.

The amount of early-apoptotic cells was expressed as the percentage of PI-negative/annexin V-positive cells (Figure 3(a), lower right quadrant of each plot), and the amount of late-apoptotic cells was expressed as the percentage of PI-positive/annexin V-positive cells (Figure 3(a), upper right quadrant of each plot). VAN (3 and 6 mg/mL) caused an increase in the percentage of both early-apoptotic and late-apoptotic cells (Figures 3(b) and 3(c)). Cilastatin significantly reduced this increase in both early and late-apoptotic cells (Figures 3(b) and 3(c)).

### 3.4. Cilastatin Downgrades VAN-Induced Mitochondrial Damage

**Figure 4:**
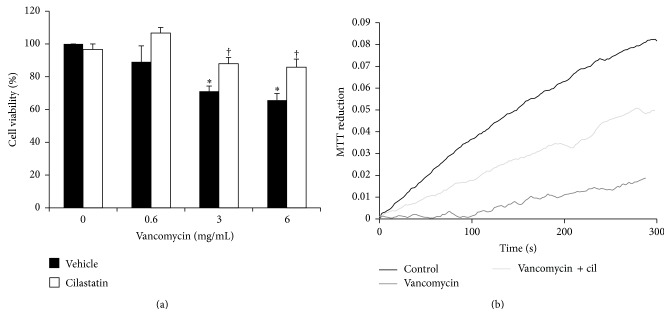
Effect of cilastatin on vancomycin-induced mitochondrial damage. Renal proximal tubular epithelial cells (RPTECs) were exposed to vancomycin and vancomycin plus cilastatin (200 *μ*g/mL) for 24 hours. (a) Cell viability was determined by the ability to reduce MTT. Results are expressed as the percentage of the value obtained relative to control (without vancomycin and cilastatin) of at least three separate experiments. (b) Changes in the mitochondrial oxidative capacity of RPTECs were assessed by MTT reduction at 570 nm. The graph shows formation of formazan as detected in isolated cells in real time with no treatment (control) and vancomycin 6 mg/mL with or without 200 *μ*g/mL cilastatin, after the incubation times in seconds given on the *x*-axis. ^*∗*^ *p* < 0.05 versus control and control plus cilastatin, ^†^ *p* < 0.05 versus the same data without cilastatin.

We quantified the functional impact of treatment with VAN on cell survival by measuring the percentage of adherent cells still able to reduce MTT to formazan after exposure to increasing doses of VAN. Coincubation with cilastatin increases cell survival in every condition analyzed. Differences that were statistically significant were only found for incubations with cilastatin in VAN 3 and 6 mg/mL for 24 h (Figure 4(a)).

Moreover, the effect of VAN on mitochondria was observed very early after addition of VAN to cell culture plates. In Figure 4(b), an inverted IX-80 microscope was fitted to obtain absorbance readings at specific wavelengths on single (or small groups of) cells in culture.

A quick and deep depression in MTT reduction activity was observed in RPTECs exposed to VAN 6 mg/mL compared with controls (Figure 4(b)). Coincubation with cilastatin partially recovers this effect. Differences are observed even during the first 5 minutes after addition of VAN.

### 3.5. Cilastatin Improves Long-Term Recovery and Cell Viability in RPTECs after Exposure to VAN

**Figure 5:**
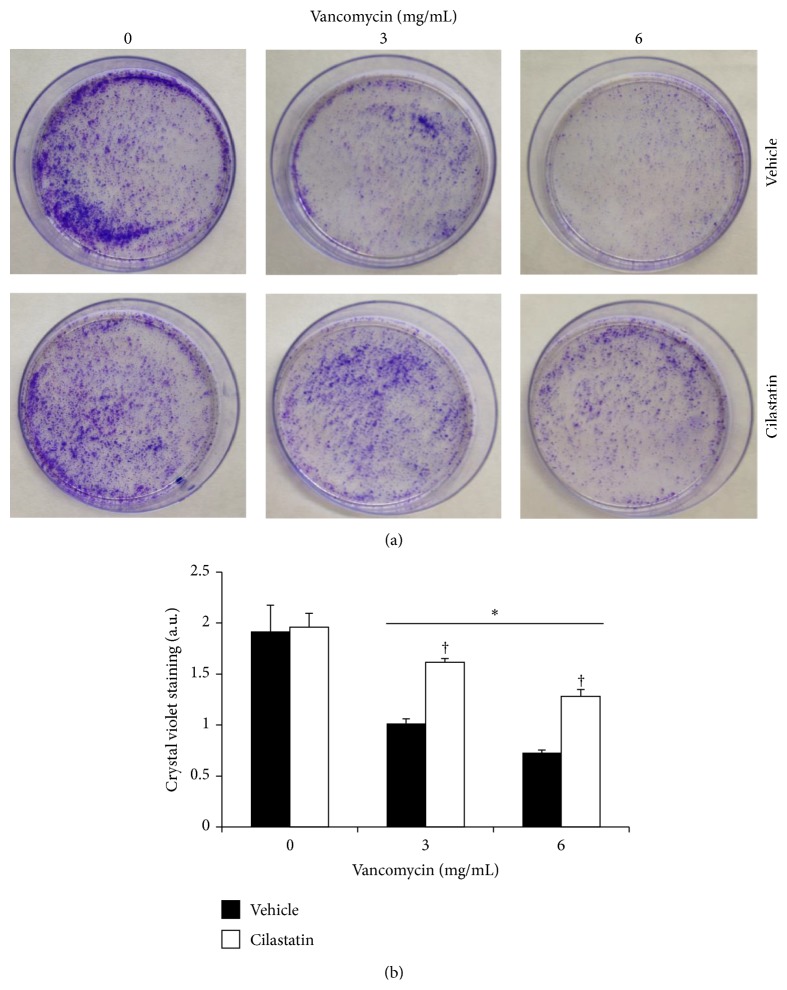
Cilastatin preserves long-term recovery of vancomycin-treated proximal tubular epithelial cells (RPTECs). (a) RPTECs were incubated with vancomycin 3 and 6 mg/mL in the presence or absence of 200 *μ*g/mL cilastatin for 24 hours. The number of colony-forming units was determined by staining with crystal violet after 7 days. (b) Quantification of crystal violet staining. Data are expressed as mean ± SEM of three separate experiments. ^*∗*^ *p* < 0.05 versus control and control plus cilastatin, ^†^ *p* < 0.05 versus the same data without cilastatin.

To know the long-term viability of surviving RPTECs after 24 hours of exposure to VAN, we tested the ability of those cells to proliferate into new cell colonies. CFUs were quantified as specified in Section 2. The CFU count decreased after 24 hours of treatment with VAN, and this decrease was clearly dose-dependent (Figure 5(a)). When VAN was exposed in the presence of cilastatin, the number of CFUs was significantly higher after 7 days of recovery for every VAN concentration studied. The intracellular dye was extracted, and absorbance was quantified at 595 nm (Figure 5(b)).

### 3.6. Cilastatin Reduces Intracellular Accumulation of VAN

**Figure 6:**
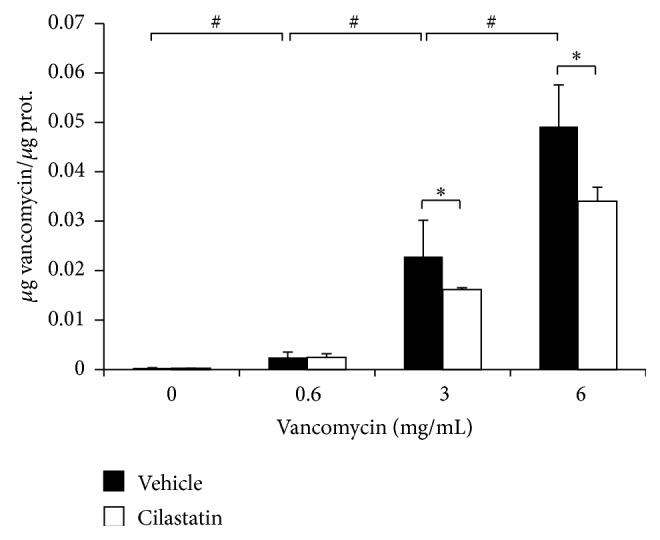
Effects of cilastatin on vancomycin accumulation in proximal tubular epithelial cells (RPTECs). Intracellular accumulation was measured in lysates of PTECs treated with vancomycin 0.6, 3, and 6 mg/mL for 24 hours, in the presence or absence of cilastatin (200 *μ*g/mL), using fluorescence polarization immunoassay (TDX) specific assays. Cilastatin was shown to prevent entry of vancomycin into RPTECs. Values were expressed as means ± SEM of vancomycin concentration (*n* = 4 different experiments). ANOVA model *p* < 0.0001. Factors: cilastatin effect ^*∗*^ *p* < 0.05; dose effect ^#^ *p* < 0.05.

In many cases, nephrotoxicity is in part dependent on the intracellular concentration of drug. As cilastatin is a ligand of the brush border membrane, we investigated whether it could affect VAN uptake by RPTECs. To test this hypothesis, we measured intracellular VAN content by TDX analysis, as described in Section 2.  Figure 6 shows that cellular VAN content increased progressively in a dose-dependent manner when RPTECs were incubated for 24 hours in the presence of different concentrations of drug. Coincubation with cilastatin significantly reduced accumulation of VAN into the cells for every concentration studied (Figure 6). These results confirm that incubation with cilastatin in primary cultures of RPTECs decreases cellular accumulation of VAN. This effect may be involved in the observed reduction of VAN impact on RPTECs damage death and survival.

### 3.7. Cilastatin Has No Effect on the Antimicrobial Action of VAN

The MICs and MBC values of VAN obtained for each isolate in the absence or with the addition of cilastatin were either the same or varied within ±1log⁡2 dilution (Table 1), thus implying that cilastatin does not inhibit the activity of VAN against any of the isolates tested.

## 4. Discussion

CRBSI are a common complication of coronary and intensive care units. When CRBSI is caused by MRSA, then polypeptide antibiotics are the only alternative to methicillin. VAN is one of the most commonly used antibiotics in the clinical management of MRSA-induced CRBSI. Clinical and preclinical studies have shown that nephrotoxicity is the main side effect of VAN and that this in turn induces AKI, thus limiting dose and duration of administration [2, 31]. Renal impairment can also influence the prognosis of patients with cardiovascular disease, thus increasing cardiovascular risk. In fact, renal dysfunction is a major risk factor for the development of nonrenal complications and a marker of lesions elsewhere in the vascular tree [32, 33]. It is associated with increased morbidity and mortality, prolonged hospital stays, and higher healthcare costs [2, 34]. Therefore, prevention of renal dysfunction and preservation of the proximal tubule are key components in strategies aimed at preventing renal damage and potential cardiovascular complications.

Several studies have shown RPTECs to be a key target of VAN-induced toxicity [8, 11, 25, 35]. Although the pathogenesis of VAN-induced nephrotoxicity is not fully understood, several mechanisms are known to cause and amplify renal damage [1, 2, 8, 11].

In proximal tubule cell cultures, VAN concentrations similar to the observed plasma levels with therapeutic doses have shown that VAN induced apoptosis but not necrosis cell death [7, 36]. Consistent with these results, we found that direct observation of VAN-treated primary cell cultures revealed characteristic apoptotic morphological changes in a dose-dependent way. Necrosis was only observed after 48 hours of treatment, never higher than a 5%. RPTECs treated with VAN presented early and severely diminished capacity to reduce MTT to formazan, directly related to mitochondrial damage. Several authors consider alteration of mitochondrial function in RPTECs to be a major factor in VAN-induced nephrotoxicity [35, 37], leading to DNA degradation and cell death, as recently demonstrated elsewhere [7].

Previous studies have shown that VAN-induced nephrotoxicity may be alleviated* in vivo* by cilastatin (imipenem/cilastatin) simultaneous treatment. Toyoguchi et al. [25] showed that cilastatin may reduce VAN-induced nephrotoxicity in rabbits by decreasing serum BUN and creatinine levels. Nakamura et al. [26, 38] presented similar results in rats. Both authors conclude that the protection observed after treatment with cilastatin is associated with reduced accumulation of VAN in renal tubules [25, 26, 38]. In fact, accumulation of VAN in renal cells has been proposed as a major cause of toxicity [2, 35, 37]. Our results are consistent with these findings as we recorded significant reductions in the accumulation of VAN in the presence of cilastatin, although, to our knowledge, ours is the first study to demonstrate that cilastatin is able to reduce apoptosis and mitochondrial injury in RPTECs.

Some authors suggested that the mechanism behind VAN-induced renal damage was similar to that of gentamicin [10, 26, 39], which is induced by accumulation of the drug from the brush border membrane to the renal proximal tubules [40, 41]. Gentamicin is transported inside the cell by endocytosis involving megalin, a brush border lipid raft ligand [41]. VAN and gentamicin colocalize in endosomes in the renal proximal tubular cells [42] and activate cathepsins triggering apoptosis [41]. If gentamicin accumulation is reduced by inhibition of its transport mechanisms, nephrotoxicity is alleviated [41].

We have published that binding of cilastatin to lipid raft bound DHP-I inhibits any vesicle based transport or signalization requiring internalization of the brush border lipid raft in proximal tubules [19–21]. In fact, cilastatin seems to be able to reduce luminal entry of drugs across the membranes (e.g., CsA, tacrolimus, and cisplatin) even if they are not substrates for DHP-I activity [18, 19]. Although the exact mechanism of VAN accumulation in proximal cells has not been elucidated yet [36] and remains open to debate [26, 43, 44], Fujiwara et al. [42] recently revealed that significant amounts of VAN were present in the apical pole, specifically in the S1 and S2 segments of the proximal tubules. The presence of VAN near the brush border could also suggest the presence of an unknown transporter(s) in this area [42], a hypothesis that was also reported by Nakamura et al. [36]. Cilastatin seems to be able to interfere with VAN transport, as previously described for other toxins [18, 19]. Thus, interference by cilastatin with VAN uptake and accumulation on RPTECs could also explain the fast protection observed in real-time experiments performed to analyze mitochondrial oxidative capacity and integrity. VAN immediately inhibits reduction of MTT to formazan, although coincubation with cilastatin partially restores this process. The very short time course of the cilastatin blocking effect strongly suggests that cilastatin inhibits uptake of VAN by RPTECs, a process that was already described by Toyoguchi et al. [25] and Nakamura et al. [26, 38]. Interference with entry of VAN could also explain the renal protection associated with a decrease in cell death by apoptosis.

Other mechanisms could also be involved in the ability of cilastatin to protect against VAN-induced nephrotoxicity. Previous results obtained by our group showed the ability of cilastatin to inhibit apoptosis induced by other nephrotoxic agents, such as CsA, tacrolimus [19], and cisplatin* in vitro* and* in vivo* [16–18] without interfering with their effectiveness on their respective target cells. Cilastatin was able to inhibit cellular and nuclear morphological changes, mitochondrial depolarization and release of cytochrome c, caspase activation, DNA fragmentation, and cell death caused by apoptosis but not necrosis in RPTECs [18].

In our model of cisplatin-induced nephrotoxicity, cilastatin inhibits internalization of the Fas-Fas ligand system bound to cell membrane lipid rafts blocking apoptosis amplification and protecting the cells [15, 18]. We do not know if the same mechanism applies in the VAN-induced renal apoptosis, but it is clear that DHP-I binding to brush border lipid rafts on RPTECs gives cilastatin the chance to interfere with the process of apoptosis.

Interestingly, our analysis of the effect of cilastatin on VAN-sensitive bacteria showed that cilastatin did not modify the MIC or MBC of VAN against any of the isolates tested. These results were expected owing to the absence of brush border and DHP-I in bacteria, thus demonstrating a specific effect on RPTECs. We show that cilastatin has a promising therapeutic role in humans. Moreover, some authors have previously reported that treatment with imipenem/cilastatin has nephroprotective effects on CsA-induced AKI in kidney recipients [21], bone marrow recipients [22], and heart recipients [23]. Therefore, protection against kidney damage caused by VAN used to treat MRSA-induced CRBSI is possible, specifically in patients with AKI.

In conclusion, our results show that cilastatin attenuates VAN-induced acute renal failure* in vitro* by decreasing apoptosis without affecting antibacterial activity. This effect could be related, at least in part, to the reduction in accumulation of the drug in cells. Therefore, cilastatin could represent a novel therapeutic approach in reducing VAN-induced renal damage without compromising bactericidal efficacy.

## Figures and Tables

**Table 1:** *In vitro* activity of vancomycin alone and with cilastatin against clinical isolates of *Staphylococcus aureus* and *Enterococcus *spp.

	Strain 1		Strain 2		Strain 3		Strain 4	
*Staphylococcus aureus *	Vehicle	Cil	Vehicle	Cil	Vehicle	Cil	Vehicle	Cil
MIC	0.5	1	0.5	1	1	0.5	0.5	0.5

MBC	16	32	2	2	1	0.5	0.5	0.5

	Strain 5		Strain 6		Strain 7		Strain 8	

*Enterococcus* spp.	Vehicle	Cil	Vehicle	Cil	Vehicle	Cil	Vehicle	Cil
MIC	0.5	0.5	0.25	0.5	1	2	0.5	0.5

MBC	>16	>16	>4	>8	>16	>32	>8	>8

Table shows the effect of cilastatin (200 *µ*g/mL) against inhibitory and bactericidal activity of vancomycin (0–64 *µ*g/mL) in clinical bacteria isolated. *Staphylococcus aureus*: strains numbers 1 and 4, methicillin-resistant; strain numbers 2 and 3, methicillin-susceptible. *Enterococcus* spp.: strains numbers 5, 7, and 8, *E*. *faecalis*; strain number 6, *E*. *faecium*.

MIC, minimum inhibitory concentration; MBC, minimum bactericidal concentration; vehicle, cation-adjusted Mueller-Hinton broth; cil, cilastatin.
